# Supplementary material for: Individualization of Mycophenolic Acid Therapy through Pharmacogenetic, Pharmacokinetic and Pharmacodynamic Testing
Source: Biomedicines. 2022 Nov 10;10(11):2882. doi: 10.3390/biomedicines10112882 (PMC9687418; doi:10.3390/biomedicines10112882)
Supplement: Supplementary file 1 [file biomedicines-10-02882-s001.zip › biomedicines-2000940-supplementary.pdf]

Supplementary Files:

Supplementary Table S1. 96-well plate pipetting scheme of the IMPDH assay. Numbers 1 to 40 indicate sample positions.

	**t0**						**t180**					
	1	2	3	4	5	6	7	8	9	10	11	12
A	S1-XMP	1	9	17	25	33	S1-XMP	1	9	17	25	33
B	S2-XMP	2	10	18	26	34	S2-XMP	2	10	18	26	34
C	S3-XMP	3	11	19	27	35	S3-XMP	3	11	19	27	35
D	S4-XMP	4	12	20	28	36	S4-XMP	4	12	20	28	36
E	S5-XMP	5	13	21	29	37	S5-XMP	5	13	21	29	37
F	S6-XMP	6	14	22	30	38	S6-XMP	6	14	22	30	38
G	AQ	7	15	23	31	39	AQ	7	15	23	31	39
H	QC	8	16	24	32	40	QC	8	16	24	32	40

Abbreviations: AQ, high performance liquid chromatography grade water; QC, quality control; XMP, xanthosine 5´-monophosphate.

Supplementary Table S2. Biopsy Results of Study Patients.

**Pathological biopsy result**	
- Borderline lesion	51
**Graft rejections:**	
**T cell-mediated rejection (TCMR):**	(33)
- Ia	8
- Ib	2
- IIa	14
- IIb	6
- chronic active TCMR	3
**Antibody-mediated rejection (ABMR):**	(8)
- acute/active ABMR	4
- chronic, active ABMR	4

Abbreviations: ABMR, antibody-mediated rejection; TCMR, T cell-mediated rejection.
